# Endothelial Dysfunction and Obesity: New Diagnostic and Therapeutic Strategies

**DOI:** 10.3390/ijms27177552

**Published:** 2026-08-24

**Authors:** Rosaria Vincenza Giglio, Sanja Stankovic, Angelo Maria Patti, Manfredi Rizzo, Marcello Ciaccio

**Affiliations:** 1 Department of Biomedicine, Neurosciences and Advanced Diagnostics, Institute of Clinical Biochemistry, Clinical Molecular Medicine and Clinical Laboratory Medicine, University of Palermo, 90127 Palermo, Italy; rosariavincenza.giglio@unipa.it (R.V.G.); marcello.ciaccio@unipa.it (M.C.); 2 Center for Medical Biochemistry, University Clinical Center of Serbia, 11 000 Belgrade, Serbia; 3 Faculty of Medical Sciences, University of Kragujevac, 34 000 Kragujevac, Serbia; 4 Department of Health Promotion Sciences, Maternal and Infant Care, Internal Medicine and Medical Specialties, University of Palermo, 90127 Palermo, Italy; pattiangelomaria@gmail.com (A.M.P.); manfredi.rizzo@unipa.it (M.R.); 5 Department of Laboratory Medicine, University Hospital “Paolo Giaccone”, 90127 Palermo, Italy

**Keywords:** obesity, endothelial dysfunction, cardiovascular risk, vascular inflammation, oxidative stress, insulin resistance, GLP-1 receptor agonists, SGLT2 inhibitors, lifestyle intervention, precision medicine

## Abstract

Endothelial dysfunction is a key mechanism linking obesity, metabolic disturbances, and cardiovascular disease, contributing to the development and progression of atherosclerosis and other vascular complications. This review provides a comprehensive overview of the molecular mechanisms underlying endothelial dysfunction in obesity and discusses current diagnostic approaches and therapeutic strategies aimed at restoring vascular homeostasis. The available evidence indicates that chronic inflammation, oxidative stress, insulin resistance, reduced nitric oxide bioavailability, increased reactive oxygen species production, and dysregulated adipokine signaling play central roles in endothelial impairment. Recent advances in functional vascular assessment, circulating biomarkers, and imaging techniques have improved the early identification of endothelial dysfunction and cardiovascular risk. Current therapeutic strategies include pharmacological agents, such as glucagon-like peptide-1 receptor agonists, sodium-glucose co-transporter 2 inhibitors, metformin, and dipeptidyl peptidase-4 inhibitors, together with lifestyle interventions based on healthy dietary patterns and regular aerobic and resistance exercise. These approaches improve glycemic control, reduce inflammation and oxidative stress, enhance endothelial function, and contribute to cardiovascular protection. Overall, the evidence supports an integrated and personalized management strategy targeting both metabolic and vascular abnormalities to reduce cardiovascular risk and improve long-term clinical outcomes in individuals with obesity.

## 1. Introduction

Obesity represents a major global health challenge and a leading risk factor for metabolic and cardiovascular diseases, including type 2 diabetes mellitus (T2DM). Endothelial dysfunction is one of the earliest vascular alterations associated with obesity and represents a key link between metabolic disturbances, insulin resistance, and atherosclerosis. Adipose tissue expansion promotes immune cell infiltration, chronic inflammation, oxidative stress, and alterations in vascular homeostasis. Adipocytes function as active endocrine cells, releasing cytokines, chemokines, and hormones collectively known as adipokines, which regulate metabolic and vascular responses [1]. The vascular endothelium is a highly specialized and heterogeneous cellular layer whose structure and function vary across different vascular beds and tissues. Based on their morphology and permeability characteristics, the endothelium can be broadly classified into continuous, fenestrated, and sinusoidal types. Continuous endothelium is characterized by closely apposed endothelial cells and is predominant in tissues such as skeletal muscle, heart, and lung; fenestrated endothelium contains specialized pores that facilitate the exchange of solutes and macromolecules and is found mainly in organs involved in filtration and secretion, such as the kidney and endocrine glands. Sinusoidal endothelium is characterized by larger intercellular gaps and a discontinuous basement membrane, allowing greater permeability, and is found in organs such as the liver, spleen, and bone marrow. Despite these structural differences, endothelial cells share a central role in maintaining vascular homeostasis through the regulation of vascular permeability, vascular tone, and inflammatory responses. Alterations in these functions are central to the development of endothelial dysfunction in obesity [2,3].

Obesity-induced adipose tissue dysfunction is characterized by adipocyte hypertrophy, macrophage recruitment, and an imbalance between pro-inflammatory and protective mediators [4]. Increased secretion of tumor necrosis factor (TNF)-α, interleukin (IL)-6, and monocyte chemoattractant protein-1 (MCP-1), together with reduced adiponectin production, promotes endothelial activation, oxidative stress, and impaired nitric oxide (NO) signaling [5,6,7,8,9]. Insulin resistance and elevated circulating free fatty acids (FFAs) further contribute to endothelial injury by activating inflammatory pathways and increasing reactive oxygen species (ROS) production [10].

Perivascular adipose tissue (PVAT) plays a particularly important role in vascular regulation because of its direct interaction with the vascular wall. Under physiological conditions, PVAT releases vasoprotective mediators; however, obesity induces a dysfunctional PVAT phenotype characterized by inflammation, altered adipokine secretion, oxidative stress, and reduced endothelial nitric oxide synthase (eNOS) activity [11,12]. These alterations contribute to vascular insulin resistance, endothelial dysfunction, and accelerated atherosclerotic remodeling [13,14,15].

Recent advances have expanded the understanding of obesity-related endothelial dysfunction through the identification of novel molecular biomarkers, including inflammatory mediators, adipokines, and epigenetic regulators such as microribonucleic acids (miRNAs). In parallel, improvements in vascular functional assessment, imaging techniques, and targeted therapies have provided new opportunities for early diagnosis and personalized intervention.

**Figure 1:**
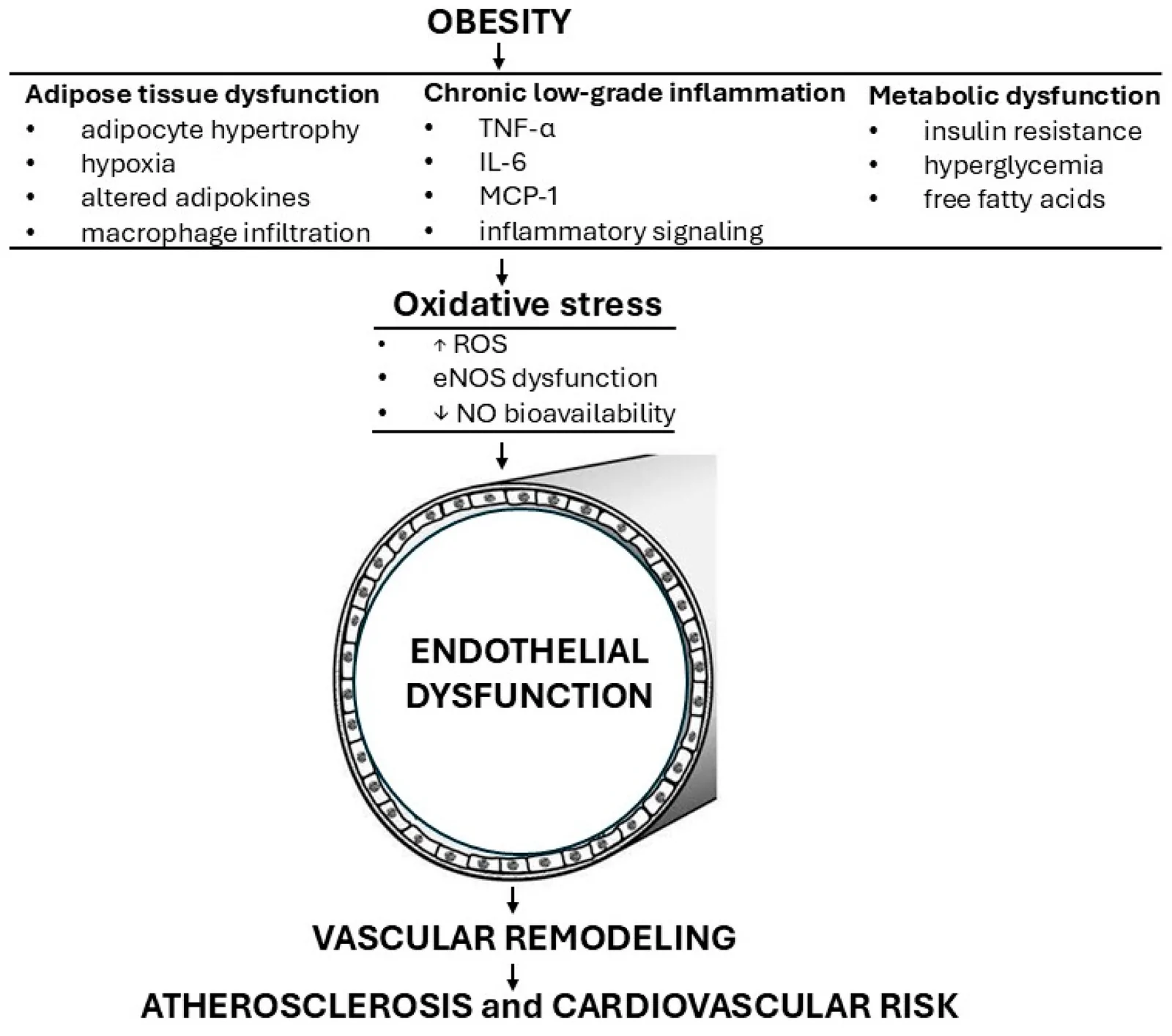
Cellular and molecular mechanisms linking obesity to endothelial dysfunction. Abbreviations: IL-6, Interleukin-6; MCP-1, Monocyte chemoattractant protein-1; NO, Nitric oxide; eNOS, endothelial nitric oxide synthase; ROS, Reactive oxygen species; TNF-α, Tumor necrosis factor-alpha.

This review summarizes the current knowledge on the molecular mechanisms linking obesity to endothelial dysfunction, with particular emphasis on emerging biomarkers, diagnostic approaches, and therapeutic strategies targeting vascular and metabolic abnormalities. A schematic overview of the main molecular and cellular mechanisms linking obesity to endothelial dysfunction is provided in Figure 1.

## 2. Adiposopathy and Endothelial Dysfunction

Obesity is a major risk factor for atherosclerosis, cardiovascular disease (CVD), and increased mortality, with the global rise in obesity prevalence paralleling the growing burden of cardiovascular complications [16,17]. Adipose tissue inflammation and insulin resistance are initiated by macrophage recruitment, chemokine activation, and increased production of pro-inflammatory adipocytokines. These alterations promote endothelial dysfunction through interconnected mechanisms, including oxidative stress, dysregulated adipokine signaling, renin–angiotensin–aldosterone system (RAAS) activation, and impaired insulin signaling [18]. The major molecular pathways linking adiposopathy to vascular dysfunction, their pathological consequences, and potential therapeutic targets are summarized in Table 1.

The coexistence of insulin resistance, dyslipidemia, and hypertension, which is commonly observed in obesity, defines a metabolic cardiorenal syndrome associated with increased cardiovascular risk. Excess energy storage in white adipose tissue promotes adipocyte hypertrophy, particularly in visceral adipose depots, which exhibit increased lipolytic activity and contribute to elevated circulating FFA levels [19,20,21]. In insulin-resistant states, impaired insulin-mediated inhibition of lipolysis further enhances FFA release, promoting oxidative stress, inflammation, and endothelial dysfunction [22].

Skeletal muscle represents the major site of insulin-stimulated glucose uptake and is therefore central to whole-body glucose homeostasis. Endothelial dysfunction may contribute to insulin resistance by impairing insulin delivery and microvascular recruitment in skeletal muscle, thereby limiting insulin-stimulated glucose uptake. This vascular component of insulin resistance further links endothelial dysfunction with metabolic impairment in obesity [23,24].

Elevated FFAs represent a key link between adipose tissue dysfunction and vascular injury by impairing insulin signaling, reducing NO bioavailability, and accelerating atherosclerotic processes [25,26]. Atherosclerosis is initiated by the accumulation of apolipoprotein B (ApoB)-containing lipoproteins within the arterial wall, a process amplified by chronic inflammation and metabolic disturbances [27,28]. These alterations contribute to vascular remodeling and increased arterial stiffness, an established marker of vascular aging and cardiovascular risk [29].

**Figure 2:**
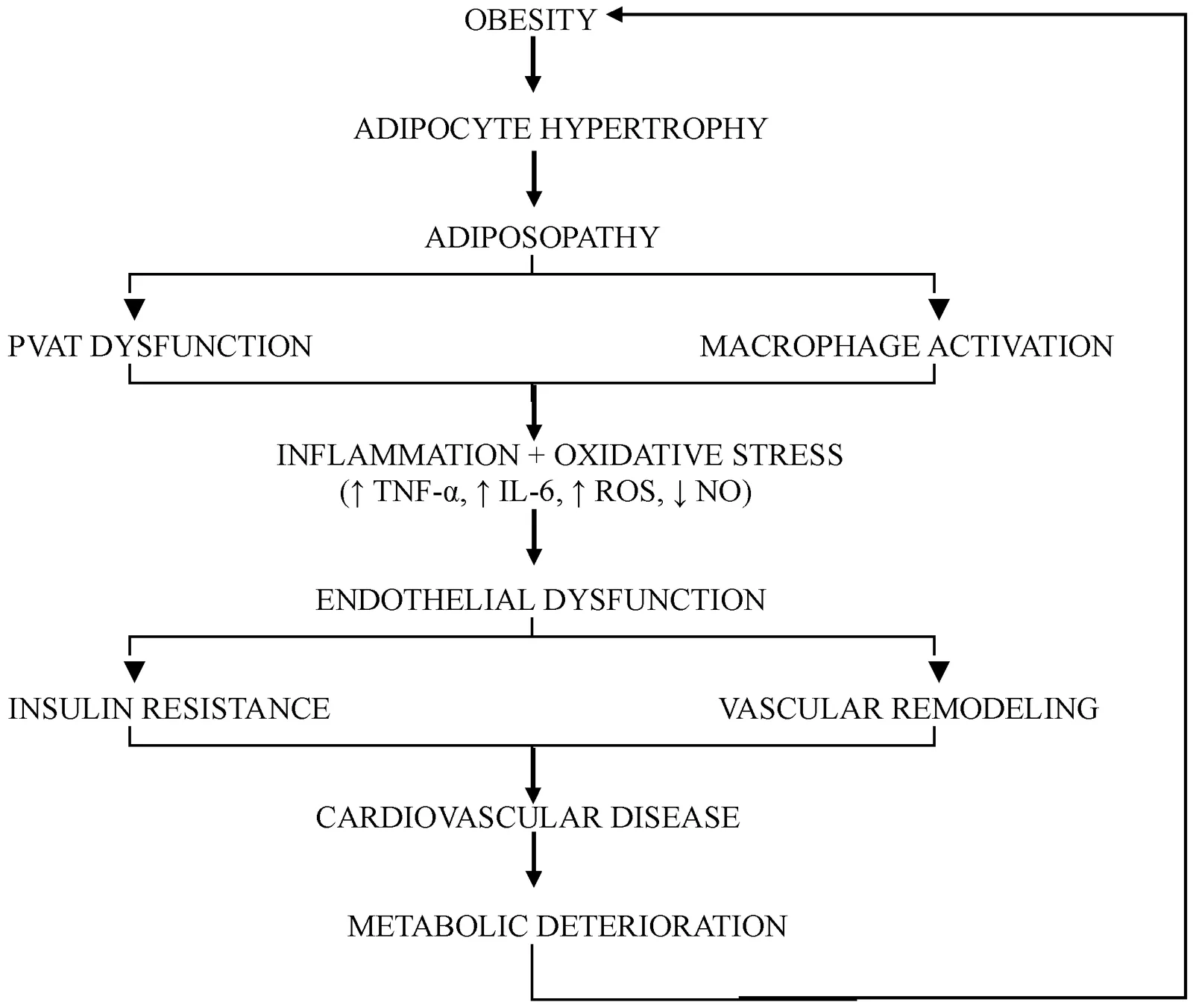
Obesity-induced adiposopathy and endothelial dysfunction: a self-amplifying pathological cycle linking inflammation, oxidative stress, and cardiovascular disease. Abbreviations: IL-6, Interleukin-6; NO, Nitric oxide; PVAT, PeriVascular adipose tissue; ROS, Reactive oxygen species; TNF-α, Tumor necrosis factor-alpha.

Although the mechanisms linking visceral obesity, inflammation, and vascular remodeling are complex, current evidence indicates that adipose tissue dysfunction, oxidative stress, and endothelial impairment interact in a self-perpetuating cycle that promotes metabolic and cardiovascular disease progression [30]. This relationship highlights the importance of targeting both metabolic and vascular pathways for early prevention and personalized therapeutic strategies (Figure 2).

## 3. Diagnostic Strategies

### 3.1. Evaluation of Flow-Mediated Dilation

Flow-mediated dilation (FMD), is a widely used non-invasive approach for assessing endothelial function and vascular health. This technique evaluates the capacity of conduit arteries, typically the brachial artery, to dilate in response to increased shear stress following transient blood flow occlusion [31,32]. After cuff release, reactive hyperemia stimulates endothelial mechanotransduction pathways, promoting eNOS activation, NO release, and subsequent vasodilation. Therefore, the magnitude of FMD reflects endothelial integrity and NO bioavailability, with impaired responses representing an early marker of vascular dysfunction and atherosclerotic risk.

Reduced FMD is frequently observed in individuals with obesity, insulin resistance, and metabolic syndrome, indicating early impairment of vascular homeostasis before overt cardiovascular disease develops [33]. Endothelium-independent assessment using nitroglycerin-mediated dilation can complement FMD measurements by evaluating vascular smooth muscle responsiveness independently of endothelial signaling [34]. Recent methodological improvements, including high-resolution ultrasound imaging, automated edge-detection systems, and standardized acquisition protocols, have enhanced reproducibility and reduced operator variability [35].

Beyond its diagnostic value, impaired FMD is associated with increased cardiovascular risk and may be useful for monitoring responses to interventions aimed at improving endothelial function [36]. Combining FMD with circulating biomarkers of endothelial activation, including IL-6, CRP, TNF-α, and asymmetric dimethylarginine (ADMA), provides a more comprehensive evaluation of vascular dysfunction and supports individualized cardiovascular risk stratification in obesity [37,38].

### 3.2. Intra-Arterial Infusion

Intra-arterial infusion represents an invasive but highly specific method for investigating endothelial function through the local administration of vasoactive agents directly into a conduit artery, such as the brachial or coronary artery. This approach commonly uses acetylcholine infusion to evaluate endothelium-dependent vasodilation mediated by NO signaling [39,40]. The endothelial response can be distinguished from vascular smooth muscle reactivity by using endothelium-independent vasodilators, such as sodium nitroprusside [41,42].

By delivering pharmacological agents at low, localized concentrations, intra-arterial infusion minimizes systemic effects and enables detailed characterization of endothelial signaling pathways. This makes it particularly valuable for mechanistic studies investigating early vascular dysfunction, endothelial molecular responses, and pharmacological modulation in high-risk populations, including individuals with obesity and insulin resistance [35,36].

Despite its high sensitivity, this methodology requires specialized expertise, strict protocol standardization, and careful monitoring of potential complications, including local vasospasm and vascular injury [43]. Due to its invasive nature, intra-arterial infusion is mainly applied in research settings rather than routine clinical assessment.

### 3.3. Laboratory Tests and Biomarker Assessment

#### 3.3.1. Inflammatory and Lipid Biomarkers

Perivascular adipose tissue (PVAT) represents an active endocrine organ that regulates vascular homeostasis through adipokine secretion, inflammatory signaling, oxidative stress modulation, and epigenetic mechanisms. In obesity, adipokine imbalance, insulin resistance, dietary excess, and altered miRNAs expression contribute to endothelial dysfunction and accelerate atherosclerotic processes. Therefore, circulating biomarkers reflecting inflammation, lipid abnormalities, and endothelial injury could be valuable tools for early detection and cardiovascular risk stratification.

Among inflammatory mediators, IL-6 plays a central role in obesity-related endothelial dysfunction by activating inflammatory pathways, stimulating hepatic C-reactive protein (CRP) production, enhancing monocyte recruitment, and promoting vascular remodeling [44,45,46]. High-sensitivity CRP (hs-CRP) levels reflect low-grade systemic inflammation and correlates with obesity severity, insulin resistance, and impaired endothelial function [47]. Ferritin, an acute-phase protein involved in iron storage and homeostasis, may also serve as a marker of chronic low-grade inflammation and metabolic dysfunction. Elevated circulating ferritin levels have been associated with obesity, adipose tissue dysfunction, insulin resistance, and features of the metabolic syndrome [48]. In obesity, increased ferritin may reflect both inflammatory activation and alterations in iron homeostasis, potentially contributing to oxidative stress and metabolic impairment. Therefore, ferritin may represent a complementary biomarker for the assessment of inflammation and cardiometabolic risk in individuals with obesity [49].

Additional biomarkers, including serum amyloid A (SAA), soluble intercellular adhesion molecule-1 (sICAM-1), and soluble vascular cell adhesion molecule-1 (sVCAM-1), indicate endothelial activation and increased leukocyte–endothelium interaction mediated by NF-κB signaling [50,51].

Tumor necrosis factor-alpha (TNF-α) and IL-6 represent major inflammatory drivers of endothelial dysfunction, impairing insulin signaling, reducing eNOS activity, and decreasing NO bioavailability [52,53]. The components of these pathways have potential as both biomarkers and therapeutic targets.

#### 3.3.2. Lipoprotein(a) and Advanced Lipid Profiling

Beyond conventional lipid parameters, lipoprotein(a) [Lp(a)] has emerged as an independent cardiovascular risk biomarker. Lp(a) consists of an LDL-like particle linked to apolipoprotein(a), which promotes atherogenesis through inducing oxidized phospholipid accumulation, vascular inflammation, endothelial activation, and pro-thrombotic signaling [54]. Unlike LDL cholesterol, circulating Lp(a) levels are predominantly genetically determined and remain relatively stable over time [55].

Elevated Lp(a) concentrations are associated with accelerated atherosclerosis and increased cardiovascular risk. In obesity, Lp(a)-mediated endothelial injury may occur through enhanced oxidative stress, inflammatory activation and impaired vascular repair mechanisms. Therefore, Lp(a) assessment may improve cardiovascular risk stratification, particularly in individuals with metabolic abnormalities or unexplained vascular risk [56]. Novel antisense oligonucleotide and small interfering RNA (siRNA)-based therapies targeting Lp(a) synthesis are currently under investigation [57,58].

#### 3.3.3. Emerging Molecular Biomarkers and miRNAs

Microribonucleic acids (miRNAs) have emerged as important regulators of endothelial homeostasis and potential biomarkers of obesity-associated vascular dysfunction. These non-coding RNAs regulate endothelial survival, angiogenesis, inflammation, oxidative stress responses, and eNOS expression through post-transcriptional mechanisms.

Several miRNAs have been associated with endothelial dysfunction in obesity and cardiovascular disease. The expression of miRNA-126, a key regulator of endothelial repair and angiogenesis, is frequently reduced in vascular disease, whereas increased expression of miRNA-21 and miRNA-92a has been linked to endothelial inflammation, impaired regeneration, and vascular remodeling [59]. Other miRNAs involved in adipogenesis and insulin signaling, such as miRNA-103, miRNA-143, and miRNA-221, represent promising candidates for both future diagnostics and therapeutics [60,61].

Although clinical implementation remains limited by methodological standardization, profiles of circulating miRNAs may provide novel approaches for the early detection of endothelial dysfunction and precision cardiovascular risk assessment.

#### 3.3.4. Clinical Implications and Future Perspectives

The integration of inflammatory, lipid, and molecular biomarkers may improve current cardiovascular risk assessment models, which do not fully capture obesity-related endothelial inflammation and oxidative stress. Multi-marker approaches combining hs-CRP, IL-6, TNF-α, Lp(a), adhesion molecules, and circulating miRNAs may enable more accurate patient stratification and personalized interventions.

Future strategies should integrate biomarker profiling with vascular imaging, functional endothelial assessments, and artificial intelligence-based predictive models to develop comprehensive precision medicine approaches for obesity-related cardiovascular disease.

### 3.4. Imaging Techniques

Imaging techniques provide complementary information for the assessment of endothelial dysfunction, vascular remodeling, and subclinical atherosclerosis in individuals with obesity. Non-invasive approaches, including carotid ultrasound, Doppler-based vascular assessment, cardiac magnetic resonance imaging (MRI), computed tomography (CT), and positron emission tomography (PET), enable the evaluation of both structural vascular alterations and functional changes associated with endothelial injury [62].

Carotid intima-media thickness (CIMT) measurement and ultrasound plaque characterization allow for the early identification of vascular remodeling and atherosclerotic changes before the onset of clinical cardiovascular disease [63]. Ultrasound-based FMD of the brachial artery remains a widely used functional method for assessing endothelial-dependent vasodilation, reflecting NO bioavailability and eNOS activity.

**Figure 3:**
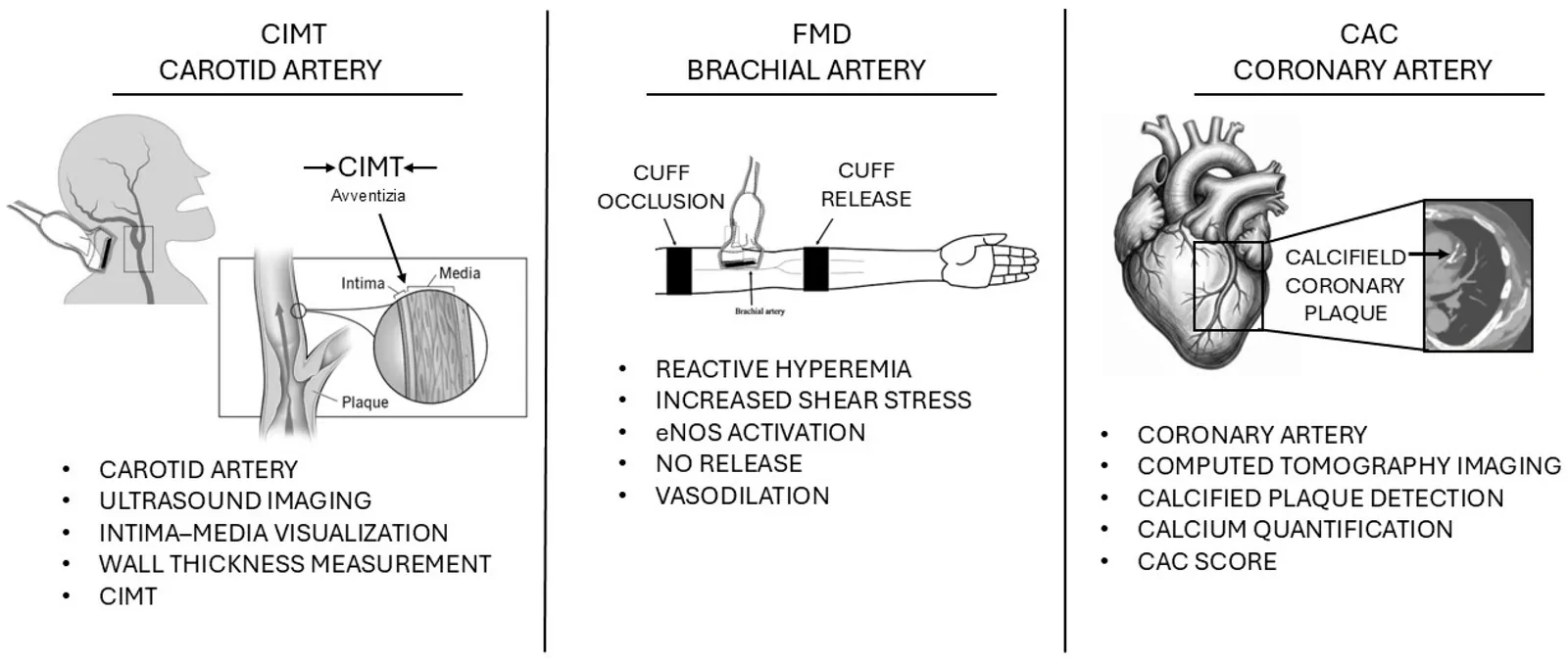
Overview of CIMT, FMD, and CAC assessments for the evaluation of vascular remodeling, endothelial function, and subclinical coronary atherosclerosis. Abbreviations: CAC, Coronary Artery Calcium; CIMT, Carotid Intima-Media Thickness; eNOS, endothelial Nitric Oxide Synthase; FMD, Flow-Mediated Dilation; NO, Nitric Oxide.

Cardiac MRI provides detailed assessment of myocardial perfusion, tissue composition, and vascular integrity without ionizing radiation exposure, offering valuable information on obesity-related cardiovascular remodeling [64]. CT-based coronary artery calcium (CAC) scoring enables quantification of calcified plaque burden and improves cardiovascular risk stratification beyond traditional lipid parameters [65]. PET imaging provides molecular insights into vascular inflammation by detecting increased metabolic activity within atherosclerotic plaques to identify potentially unstable lesions associated with adverse cardiovascular outcomes [66]. An overview of CIMT, FMD, and CAC assessments is provided in Figure 3.

In obesity, excessive accumulation of PVAT may interfere with vascular imaging interpretation while contributing directly to endothelial inflammation through adipokine dysregulation and oxidative stress pathways [67]. Therefore, advanced imaging approaches combined with circulating biomarkers, including inflammatory mediators and molecular markers such as miRNAs, may provide a more comprehensive assessment of endothelial dysfunction.

Emerging hybrid techniques, including PET/CT and PET/MRI, integrate anatomical and molecular information, allowing for the simultaneous evaluation of plaque morphology, inflammatory activity, and vascular remodeling. Furthermore, artificial intelligence-based imaging analysis may improve automated detection of subtle vascular alterations and support personalized cardiovascular risk prediction [68]. Overall, the integration of imaging modalities with molecular biomarker profiling represents a promising strategy for early diagnosis, risk stratification, and individualized management of obesity-related endothelial dysfunction.

## 4. Therapeutic Strategies

### 4.1. Pharmacological Strategies

Pharmacological strategies targeting obesity-related endothelial dysfunction aim to improve metabolic control while directly modulating vascular inflammation, oxidative stress, and endothelial signaling pathways. Among these approaches, glucagon-like peptide-1 receptor agonists (GLP-1 RAs), including liraglutide and semaglutide, have demonstrated relevant endothelial and cardiovascular protective effects [69,70,71,72,73]. These agents activate intracellular pathways involving AMP-activated protein kinase (AMPK), cyclic adenosine monophosphate (cAMP), and protein kinase A (PKA), promoting eNOS activation, increasing NO bioavailability, and reducing inflammatory signaling within vascular and perivascular adipose tissue compartments [74,75].

Dual incretin receptor agonists, such as tirzepatide, represent an emerging therapeutic class combining GLP-1 RAs and glucose-dependent insulinotropic polypeptide (GIP) signaling. Through improved metabolic regulation, reduced adipose inflammation, and enhanced vascular function, these agents may provide additional benefits in obesity-associated endothelial dysfunction [76,77].

Other metabolic therapies also exert endothelial-protective effects. Metformin improves vascular function through AMPK activation, reduction of oxidative stress, and enhancement of NO signaling [78]. Dipeptidyl peptidase-4 inhibitors (DPP-4 inhibitors), such as vildagliptin, may improve endothelial responsiveness by reducing ROS production and enhancing microvascular function [79]. Sodium-glucose cotransporter-2 inhibitors (SGLT2is), including empagliflozin, canagliflozin, and dapagliflozin, exert cardiovascular benefits through mechanisms involving reduced glucotoxicity, improved mitochondrial function, attenuation of endothelial inflammation, and preservation of NO signaling [80].

Despite these advances, no single pharmacological intervention completely reverses the complex interaction between obesity, inflammation, metabolic dysfunction, and vascular injury. Future therapeutic approaches will likely require precision strategies integrating molecular biomarkers, including inflammatory mediators and endothelial dysfunction markers, to identify patients most likely to benefit from targeted interventions. Combination approaches involving metabolic therapies, lipid-lowering agents, and anti-inflammatory strategies may provide complementary vascular protection [81,82].

### 4.2. Non-Pharmacological Strategies

Lifestyle interventions remain fundamental strategies for improving endothelial function and reducing cardiovascular risk in obesity. Dietary patterns characterized by high consumption of fruits, vegetables, whole grains, legumes, nuts, unsaturated fats, polyphenols, and omega-3 fatty acids promote vascular health by reducing oxidative stress, suppressing inflammatory pathways, and enhancing NO bioavailability [83,84,85,86,87]. Among these approaches, the Mediterranean diet has consistently demonstrated beneficial effects on lipid metabolism, inflammatory biomarkers, and endothelial-dependent vasodilation [88,89,90].

Regular aerobic and resistance exercise provides additional vascular benefits through increased shear stress-mediated activation of eNOS, improved insulin sensitivity, reduced visceral adiposity, and modulation of adipokine secretion [91,92,93,94,95,96]. Exercise-induced activation of AMPK and related antioxidant pathways contributes to improved endothelial homeostasis and reduced vascular inflammation.

Sleep duration and quality are increasingly recognized as important determinants of cardiovascular and metabolic health. Sleep deprivation, insufficient sleep, and sleep fragmentation have been associated with impaired endothelial function, including reduced endothelium-dependent vasodilation. Potential mechanisms include increased oxidative stress, systemic inflammation, autonomic imbalance, and reduced nitric oxide bioavailability, which may collectively impair endothelial homeostasis. These alterations may be particularly relevant in obesity, where sleep disturbances frequently coexist with insulin resistance, chronic low-grade inflammation, and metabolic dysfunction. Therefore, ensuring adequate and restorative sleep should be considered an important component of lifestyle interventions aimed at preserving endothelial function and reducing cardiovascular risk [97,98,99].

The combination of nutritional optimization, structured physical activity, and adequate sleep may provide complementary and potentially synergistic benefits for endothelial function and metabolic health. Furthermore, integrating lifestyle interventions with monitoring of biomarkers, including CRP, IL-6, TNF-α, and other molecular indicators, may facilitate individualized prevention strategies in high-risk obese populations [100,101,102].

### 4.3. Integrated Approach

Given the multifactorial nature of obesity-related endothelial dysfunction, integrated approaches combining pharmacological therapies, lifestyle modification, and biomarker-guided strategies represent the most promising framework for cardiovascular prevention. GLP-1 receptor agonists, SGLT2 inhibitors, metformin, and lifestyle interventions converge on common molecular mechanisms, including reduction of oxidative stress, inhibition of inflammatory signaling, restoration of eNOS activity, and improvement of metabolic homeostasis [103,104].

The incorporation of molecular biomarkers and functional vascular assessments may enable improved patient stratification and personalized treatment selection. A summary of the current diagnostic approaches and therapeutic strategies targeting obesity-related endothelial dysfunction, including vascular assessments, emerging biomarkers, pharmacological interventions, and lifestyle modifications, is provided in Table 2.

Lifestyle interventions provide complementary benefits by modulating environmental and behavioral determinants of vascular health. Diets rich in fruits, vegetables, whole grains, unsaturated fats, polyphenols, and omega-3 fatty acids improve NO bioavailability, reduce oxidative stress, and attenuate chronic inflammation, whereas reduced intake of saturated fats and added sugars contributes to cardiovascular risk reduction [83,84,88]. Regular aerobic and resistance exercise enhances endothelial function through increased shear stress-mediated activation of eNOS, improved insulin sensitivity, reduced visceral adiposity, and modulation of adipokine signaling [91,92,94,95,115].

The combination of pharmacological and lifestyle interventions represents the most effective strategy for targeting the multifactorial mechanisms underlying obesity-related endothelial dysfunction [116]. Integrated approaches improve vascular function, metabolic control, body composition, and inflammatory profiles more effectively than isolated interventions [117]. Furthermore, precision-based strategies incorporating biomarker profiling, functional vascular assessment, and patient-specific metabolic characteristics may optimize therapeutic selection and enhance endothelial protection [101,103].

By simultaneously addressing obesity, insulin resistance, oxidative stress, inflammation, and endothelial impairment, combined interventions provide a multidimensional framework for cardiovascular prevention. This approach reflects the transition toward precision cardiovascular medicine, in which treatment decisions are guided not only by traditional risk factors but also by molecular biomarkers and vascular functional parameters [83,94].

## 5. Conclusions

Endothelial dysfunction represents a central mechanism linking obesity, metabolic alterations, and cardiovascular disease. Chronic inflammation, oxidative stress, insulin resistance, and adipose tissue dysfunction contribute to impaired endothelial homeostasis, promoting vascular remodeling and atherosclerotic progression. Recent advances in functional vascular assessment, measurement of circulating biomarkers, and imaging techniques have improved the identification of early endothelial alterations, enabling more accurate cardiovascular risk stratification.

Therapeutic strategies targeting obesity-related endothelial dysfunction require a multidimensional approach that combines pharmacological interventions with lifestyle modification. Metabolic therapies, including GLP-1 receptor agonists and SGLT2 inhibitors, together with nutritional optimization and structured physical activity, improve endothelial function by modulating oxidative stress, inflammation, nitric oxide signaling, and metabolic pathways.

**Figure 4:**
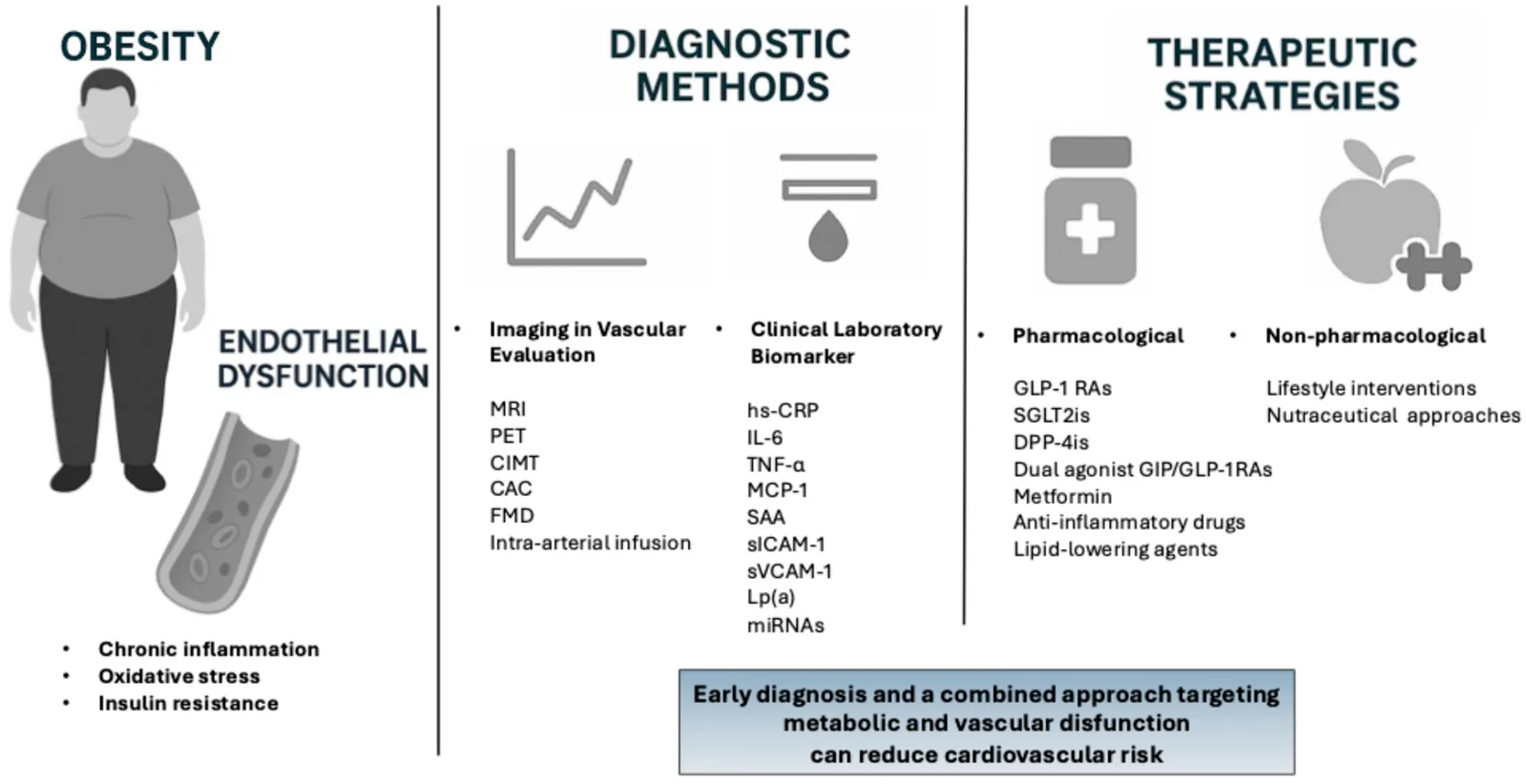
Integrated Diagnostic and Therapeutic Strategies for Endothelial Dysfunction and Obesity. Abbreviations: CAC, Coronary Artery Calcium; CIMT, Carotid Intima Media Thickness; FMD, Flow-Mediated Dilation; GIP, Glucose-dependent Insulinotropic Polypeptide; GLP-1 RAs, Glucagon-Like Peptide-1 Receptor Agonists; hs-CRP, high-sensitivity C-eactive protein; IL-6, Interleukin; Lp(a), Lipoprotein(a); MCP-1, Monocyte Chemoattractant Protein-1; miRNAs, microRiboNucleic Acids; MRI, Magnetic Resonance Imaging; PET, Positron Emission Tomography; SAA, Serum Amyloid A; SGLT2is: Sodium-Glucose Cotransporter-2 inhibitors; sICAM-1, soluble Intercellular Adhesion Molecule-1; sVCAM-1, soluble Vascular Cell Adhesion Molecule-1; TNF-α, Tumor Necrosis Factor-α.

Future approaches should integrate molecular biomarkers, functional vascular assessments, and precision medicine strategies to identify high-risk individuals and tailor interventions according to specific pathogenic mechanisms. Early, individualized, and mechanism-based management of obesity-related endothelial dysfunction may substantially reduce cardiovascular risk, improve long-term outcomes, and support a transition toward preventive cardiovascular care focused on both metabolic and vascular health. An integrated diagnostic and therapeutic approach, encompassing early vascular assessment, biomarker evaluation, lifestyle modification, and targeted pharmacological interventions, is summarized in Figure 4.

## Figures and Tables

**Table 1:** Molecular mechanisms linking obesity, adiposopathy, and endothelial dysfunction.

Pathophysiological Process	Main Molecular Mediators	Endothelial Alterations	Clinical Implications	Potential Therapeutic Targets
Adipocyte hypertrophy	Hypoxia, HIF-1α, FFAs	Adipocyte dysfunction and inflammatory activation	Obesity progression	Weight reduction, lifestyle interventions
Macrophage infiltration	MCP-1, TNF-α, IL-6	Chronic low-grade inflammation, reduced NO production	Insulin resistance	Anti-inflammatory therapies
PVAT dysfunction	↓ Adiponectin, ↑ leptin, TNF-α, IL-6, IL-8	Loss of anticontractile activity, endothelial activation	Vascular remodeling	GLP-1 RAs
Oxidative stress	ROS, NOX2, mitochondrial dysfunction	eNOS uncoupling, decreased NO bioavailability	Endothelial dysfunction	Antioxidant strategies, SGLT2is
Dyslipidemia	FFAs, oxidized LDL, ApoB lipoproteins	Foam-cell formation, endothelial activation	Atherosclerosis	Statins, PCSK9 inhibitors
Insulin resistance	Impaired PI3K/Akt pathway	Reduced eNOS phosphorylation	Impaired vasodilation	Metformin, GLP-1 RAs
RAAS activation	Angiotensin II, aldosterone	Vasoconstriction, oxidative stress	Hypertension	RAAS blockade
Chronic inflammation	IL-6, TNF-α, CRP, IL-1β	Increased VCAM-1, ICAM-1, leukocyte adhesion	Plaque progression	Anti-cytokine therapies
Arterial remodeling	Matrix metalloproteinases, collagen deposition	Increased arterial stiffness	Cardiovascular disease	Early preventive treatment

Abbreviations: ApoB, Apolipoprotein B; CRP, C-reactive protein; eNOS, endothelial nitric oxide synthase; FFAs, Free fatty acids; GLP-1 RAs, Glucagon-like peptide-1 receptor agonists; HIF-1α, Hypoxia-inducible factor-1 alpha; ICAM-1, Intercellular adhesion molecule-1; IL, Interleukin; MCP-1, Monocyte chemoattractant protein-1; NO, Nitric oxide; NOX2, Nicotinamide adenine dinucleotide phosphate (NADPH) oxidase 2; PI3K, PhosphatidylInositol 3-kinase; PVAT, Perivascular adipose tissue; RAAS, Renin–angiotensin–aldosterone system; ROS, Reactive oxygen species; SGLT2is, Sodium-gLucose cotransporter-2 inhibitors; TNF-α, Tumor necrosis factor-alpha; VCAM-1, Vascular cell adhesion molecule-1.

**Table 2:** Molecular biomarkers, diagnostic approaches, and therapeutic strategies targeting endothelial dysfunction in obesity.

Category	Method/Marker/Intervention	Mechanism and Molecular Target	Main Biomarkers or Parameters	Clinical Status and Significance	Advantages and Limitations	Key Supporting Evidence	References
**I. Molecular Biomarkers**	**ADMA**	Endogenous inhibitor of eNOS, reduces NO synthesis and endothelial-dependent vasodilation.	Plasma ADMA levels; NO bioavailability; endothelial function parameters.	Established circulating biomarker of endothelial dysfunction; elevated in obesity, insulin resistance, and metabolic disorders.	Widely validated and measurable; limited specificity because ADMA is influenced by renal function and systemic inflammation.	Clinical studies consistently demonstrate associations between elevated ADMA levels, impaired endothelial function, and increased cardiovascular risk.	[33,42,105]
	**Soluble adhesion molecules (sICAM-1, sVCAM-1, E-selectin)**	NF-κB-driven endothelial activation promotes leukocyte adhesion, vascular inflammation, and endothelial injury.	Plasma sICAM-1, sVCAM-1, E-selectin concentrations.	Established markers of endothelial inflammation and cardiovascular risk.	Easily measurable and validated; affected by acute inflammation and other systemic conditions.	Increased circulating adhesion molecules are associated with obesity, insulin resistance, and vascular inflammation.	[44,47,51]
	**ET-1**	Endothelial-derived vasoconstrictor that promotes oxidative stress, vascular smooth muscle proliferation, and vascular remodeling.	Plasma ET-1 levels; NO/ET-1 ratio.	Marker of impaired vascular homeostasis and obesity-related cardiovascular disease.	Strong biological relevance; limited specificity due to sensitivity to multiple vascular factors.	Studies demonstrate increased ET-1 activity in obesity and metabolic vascular dysfunction.	[11,33,42,106]
	**Emerging miRNAs (miRNA-126, miRNA-21, miRNA-92a, miRNA-200 family)**	Epigenetic regulation of eNOS expression, endothelial survival, angiogenesis, inflammation, and vascular repair pathways.	Profiles of circulating miRNAs; endothelial gene expression patterns.	Promising biomarkers for early diagnosis and precision cardiovascular risk stratification.	High molecular specificity; clinical application limited by lack of standardized assays.	Experimental and clinical studies support their role in endothelial dysfunction and obesity-related vascular alterations.	[59,60,61,72,107,108]
	**Adipokine imbalance (leptin-to-adiponectin ratio)**	Increased leptin activates NADPH oxidase and oxidative stress pathways; reduced adiponectin impairs anti-inflammatory and vasoprotective signaling.	Leptin/adiponectin ratio; inflammatory markers; oxidative stress markers.	Predictor of early endothelial dysfunction and cardiometabolic risk in obesity.	Simple and accessible; influenced by sex, adiposity distribution, and metabolic status.	Strong association with insulin resistance, inflammation, and vascular impairment.	[1,21,33,101]
**II. Diagnostic & Functional Approaches**	**FMD**	Measures endothelial NO-dependent vasodilation following shear stress stimulation.	Brachial artery FMD (%); NO-dependent vascular response.	Reference method for assessing macrovascular endothelial function and cardiovascular risk.	Non-invasive and prognostic; technically demanding and operator-dependent.	Extensive clinical validation demonstrates association between impaired FMD and cardiovascular outcomes.	[31,32,35,36,40,109]
	**EndoPAT**	Evaluates microvascular endothelial function through reactive hyperemia-mediated pulse amplitude changes.	RHI	Reproducible assessment of peripheral endothelial function.	Automated and less operator-dependent than FMD; limited availability.	Validated in obesity, diabetes, and cardiovascular risk populations.	[109,110]
	**PWV**	Measures arterial stiffness resulting from vascular remodeling and endothelial impairment.	Carotid-femoral PWV; arterial stiffness indices.	Clinically validated marker of vascular aging and cardiovascular risk.	Highly reproducible; indirect measure of endothelial dysfunction.	Large epidemiological studies support PWV as an independent predictor of cardiovascular events.	[29,30,111]
**III. Therapeutic Interventions**	**GLP-1 RAs (e.g., semaglutide)**	Activate AMPK signaling, enhance eNOS phosphorylation, reduce NF-κB activation, decrease oxidative stress, and preserve mitochondrial function.	FMD; NO availability; inflammatory cytokines; oxidative stress markers; HbA1c.	Provide endothelial and cardiovascular protection beyond metabolic control and weight reduction.	Strong molecular and clinical evidence; gastrointestinal adverse effects and cost may limit use.	Cardiovascular outcome trials and mechanistic studies demonstrate vascular benefits.	[69,70,71,73,75,76,78,112]
	**SGLT2is (e.g., empagliflozin)**	Reduce glucotoxicity, oxidative stress, endothelial inflammation, and NHE-1 activity; improve mitochondrial function and NO signaling.	FMD; arterial stiffness; oxidative stress markers; inflammatory biomarkers.	Improve endothelial function and reduce cardiovascular and renal events in metabolic disease.	Benefits extend beyond lowering glucose levels; effectiveness depends on clinical context.	Randomized cardiovascular outcome trials and translational studies support endothelial effects.	[59,80,103,104,113]
	**Lifestyle modification (caloric restriction and aerobic/resistance exercise)**	Activates AMPK/SIRT1 pathways, restores eNOS activity, reduces TNF-α and IL-6 signaling, and decreases mitochondrial oxidative stress.	Body weight; insulin sensitivity; FMD; inflammatory markers; oxidative stress markers.	First-line strategy for improving endothelial homeostasis and cardiometabolic health.	Safe, low-cost, and broadly applicable; long-term adherence remains challenging.	Intervention studies consistently demonstrate improvements in endothelial function and metabolic profiles.	[83,88,91,92,93,94,95,100,114]

Abbreviations: ADMA, Asymmetric DiMethylArginine; AMPK, Adenosine Monophosphate-activated Protein Kinase; Endo PAT, Endothelial Peripheral Arterial Tonometry; eNOS, endothelial Nitric Oxide Synthase; ET-1, EndoThelin-1; FMD, Flow-Mediated Dilation; GLP-1 RAs: Glucagon-Like Peptide-1 Receptor Agonists; miRNAs, microRiboNucleic Acids; NHE-1, Sodium-Hydrogen Exchanger isoform 1; NF-κB, Nuclear Factor kappa B; NO, Nitric Oxide; PWV, Pulse Wave Velocity; RHI, Reactive Hyperemia Index; SGLT2is: Sodium-Glucose Cotransporter-2 inhibitors; SIRT1, SIRTuin 1; sICAM-1, soluble InterCellular Adhesion Molecule-1; sVCAM-1, soluble Vascular Cell Adhesion Molecule-1; TNF-α, Tumor Necrosis Factor-alpha.

## Data Availability

No new data were created or analyzed in this study. Data sharing is not applicable to this article.

## Abbreviations

The following abbreviations are used in this manuscript:

AI	Artificial Intelligence
ApoB	Apolipoprotein B
ADMA	Asymmetric dimethylarginine
AMPK	AMP-activated protein kinase
ATMs	Adipose tissue macrophages
BMI	Body mass index
CAC	Coronary artery calcium
CAD	Coronary artery disease
cAMP	Cyclic adenosine monophosphate
CIMT	Carotid intima media thickness
CRP	C-reactive protein
CT	Computed tomography
CVD	Cardiovascular disease
DPP-4	Dipeptidil peptidase-4
Endo PAT	Endothelial peripheral arterial tonometry
eNOS	Endothelial nitric oxide synthase
FFAs	Free fatty acids
FMD	Flow-mediated dilation
GIP	Glucose-dependent insulinotropic polypeptide
GLP-1 RAs	Glucagon-like peptide-1 receptor agonists
HFpEF	Heart Failure with preserved ejection fraction
hs-CRP	high-sensitivity C-reactive protein
IL	Interleukin
Lp(a)	Lipoprotein(a)
LPL	Lipoprotein lipase
MCP-1	Monocyte Chemoattractant Protein-1
miRNAs	Micro ribonucleic acids
MRI	Magnetic resonance imaging
NO	Nitric oxide
NOX2	NADPH oxdase 2
PAD	Peripheral arterial disease
PET	Positron emission tomography
PKA	Protein kinase A
PVAT	Perivascular adipose tissue
RAAS	Renin–angiotensin–aldosterone system
ROS	Reactive oxygen species
SAA	Serum amyloid A
SGLT2is	Sodium-Glucose Cotransporter-2 inhibitors
sICAM-1	Soluble Intercellular Adhesion Molecule-1
sVCAM-1	Soluble Vascular Cell Adhesion Molecule-1
T2DM	Type 2 Diabetes Mellitus
TNF-α	Tumor Necrosis Factor-α
WHO	World Health Organization
